# Multi-Granularity Temporal Embedding Transformer Network for Traffic Flow Forecasting

**DOI:** 10.3390/s24248106

**Published:** 2024-12-19

**Authors:** Jiani Huang, He Yan, Qixiu Chen, Yingan Liu

**Affiliations:** College of Information Science and Technology & Artificial Intelligence, Nanjing Forestry University, Nanjing 210037, China; hjiani@njfu.edu.cn (J.H.); yanhe@njust.edu.cn (H.Y.); chenqixiu0618@njfu.edu.cn (Q.C.)

**Keywords:** traffic flow forecasting, deep learning, attention mechanism, multi-granularity embedding, spatio-temporal series

## Abstract

Traffic flow forecasting is integral to transportation to avoid traffic accidents and congestion. Due to the heterogeneous and nonlinear nature of the data, traffic flow prediction is facing challenges. Existing models only utilize plain historical data for prediction. Inadequate use of temporal information has become a key problem in current forecasting. To address the problem, we must effectively analyze the influence of time periods while integrating the distinct characteristics of traffic flow across various time granularities. This paper proposed a multi-granularity temporal embedding Transformer network, namely MGTEFormer. An embedding input adeptly merges complex temporal embeddings, a temporal encoder to consolidate rich temporal information, and a spatial encoder to discern inherent spatial characteristics between different sensors. The combined embeddings are fed into the attention mechanism’s encoder, culminating in prediction results obtained through a linear regression layer. Temporal embedding can help by fussing the period and various temporal granularities into plain historical traffic flow that can be learned adequately, reducing the loss of time information. Experimental analyses and ablation studies conducted on real traffic datasets consistently attest to the superior performance of the MGTEFormer. Our approach reduces the mean absolute error of the original models by less than 1.7%. Extensive experiments demonstrate the superiority of the proposed MGTEFormer over existing benchmarks.

## 1. Introduction

Traffic flow prediction is a crucial component of Intelligent Transportation Systems (ITSs), which are essential elements of smart cities [1]. Addressing traffic accidents and congestion is vital for improving an individual’s quality of life and safety [2]. Reliable traffic flow prediction is essential for optimizing traffic systems by providing valuable information to reduce accidents and congestion, as well as assisting transportation system managers in their traffic control efforts [3]. Traffic flow prediction serves as the core of ITS for traffic management [4,5]. However, traditional methods rarely adequately capture the complex temporal pattern [6].

In recent years, an extensive body of research has emerged in traffic flow analysis. Traffic flow prediction can be classified into three main methods: classical statistical approaches, traditional machine learning approaches, and deep learning approaches [7]. Classical statistical approaches include historical averages, auto-regressive integrated moving average [8], and Kalman filter [9], view the temporal feature solely as a time series problem [10], limiting the exploration of the traffic forecasting issue. The traditional machine learning approaches such as K-nearest neighbors [11], support vector regression [12], and neural networks have proven useful in capturing temporal trends in traffic flow. However, these methods may face challenges when dealing with large datasets, often leading to decreased performance in such scenarios.

Deep learning approaches such as CNN-RNN-based methods leverage both Convolutional Neural Networks (CNNs) and Recurrent Neural Networks (RNNs) to capture spatial and temporal dependencies, respectively. It is difficult for CNN-RNN-based methods to capture spatial features inherent in an Euclidean space [13]. GCN-based methods utilize Graph Convolutional Networks (GCNs) [14] to capture spatial correlations within non-Euclidean spaces using graph neural networks. However, plain GCN methods rely on static representations, which may not adequately capture dynamic changes in spatial behavior over time [6]. On the other hand, GAT-based methods, which employ Graph Attention Networks (GATs) [15], are designed to learn both spatial and temporal dependencies simultaneously. GATs calculate attention scores to dynamically update spatial–temporal information. Nevertheless, these methods often overlook subtle temporal features of traffic flow data, such as periodicity and temporal variations, which are essential for a comprehensive analysis of complex traffic dynamics.

Existing studies [4,14,16] have acknowledged the significant impact of temporal features on spatio-temporal dependencies. However, these methods overlook the interactions between various temporal granularities and sequences, potentially hindering the model’s ability to effectively capture spatio-temporal dependencies. Additionally, many models neglect the chronological positioning of data, such as the specific day of the week, which captures patterns like differences between weekdays and weekends. These limitations potentially hinder the models’ ability to comprehensively learn the intrinsic properties of spatio-temporal data, leading to suboptimal performance in traffic flow prediction.

To alleviate these challenges, we propose a deep learning model, Multi-Granularity Temporal Embedding Transformer (MGTEFormer), to predict traffic flow at different granularity temporal embeddings among different sensors. The Multi-Granularity Temporal Embedding (MGTE) framework designed in this work captures comprehensive temporal information by combining the position offsets of chronological characteristics with sequence samples at various granularities. This design enables the model to learn both the periodicity and positional features inherent in traffic data, providing a more detailed representation of temporal patterns. Contrary to traditional models such as space–time multivariate negative binomial regression [17] and negative binomial additive models [18], which rely on specific statistical distributions (e.g., Poisson or negative binomial) to address data variance, the proposed MGTEFormer model adapts to data variability through adaptive embeddings. The model learns from dynamic features during training by adjusting the matrix weight parameters, allowing it to capture complex temporal dependencies without relying on distributional assumptions. The attention mechanism extracts dynamic futures and alleviates future fusion. Experiments on the public dataset show that our proposed MGTEFormer can outperform other baselines. We also conducted an ablation study for a better understanding of our method. The main contributions of this paper are summarized as follows:

- We developed a multi-granularity temporal embedding for a spatial-temporal model, incorporating periodicity and chronological embeddings to capture inherent traffic flow trends.
- Two attention-based transformer encoder blocks are designed to dynamically learn temporal and spatial features on different timesteps and sensors.
- Extensive experiments are conducted on real-world traffic datasets, which verify that our model achieves the best prediction performances compared to the existing baselines.

The rest of this paper is organized as follows. In Section 2, we review the related works in traffic forecasting and different embedding designs. Section 3 presents the prediction problem formulations and a multi-granularity temporal embedding transformer network for traffic flow forecasting. In Section 4, we conduct experiments on the real-world dataset and compare prediction performance with some existing methods. The discussion is drawn in Section 5. In Section 6, we conclude this paper and give promising future work.

## 2. Related Works

This section reviews traffic flow prediction and temporal embedding, respectively. In addition, two different sections are discussed: traffic forecasting and multi-granularity temporal embedding.

### 2.1. Traffic Forecasting

In recent years, traffic flow forecasting has increasingly relied on deep learning methods to predict future traffic states using historical data [19,20], and can be split into three prominent categories: CNN-RNN-based methods, GCN-based methods, and GAT-based methods.

The CNN-RNN-based models are widely used. RNN-based models apply recursive structure. Limited by the gradient vanishing or explosion problem, Fu et al. introduced Long Short-Term Memory (LSTM) and Gated Recurrent Units (GRUs) [21], which have demonstrated high performance coupled with low computational complexity. To mitigate the significant increase in computational cost associated with WaveNet [22], there has been a shift towards Temporal Convolutional Neural Networks (TCNs) with parallel 1D-CNN structures. Additionally, to enhance the capture of longer-range temporal dependencies, Zhao et al. [23] employed 1D-CNN with dilated factors. However, they often face challenges in capturing spatial features inherent in underlying graph structures.

Considering the structure of the traffic network, researchers integrate Graph Neural Networks (GNNs), which learn spatial dependencies in non-Euclidean space [24]. In recent years, popular GNNs can be divided into three categories: spectral GCNs, spatial GCNs, and GATs [25]. ChebNet has become one of the most popular Spectral GNN methods to use a truncated expansion of Chebyshev polynomials [4]. However, they lie in their dependence on the graph Laplacian matrix. The graph Laplacian matrix must be recalculated with the change in the graph structure. Therefore, Jiang et al. [26] developed spatial GCNs to release this limitation by passing messages in the spatial domain. However, conventional GCN methods relying on static representations may fall short in capturing dynamic changes in spatial behavior over time.

GAT-based models are first proposed in the field of Natural Language Processing (NLP) [27]. The attention mechanism has revolutionized various tasks by enabling models to dynamically focus on different parts of the input data to improve performance. Yu et al. add a self-attention block on the STGCN [14] to develop the ASTGCN [28]. The Graph Multi-Attention Network (GMAN) [15], with an encoder-decoder structure, can model the effects of spatial-temporal factors on traffic conditions. Considering the dynamic spatial independence, Guo et al. [29] propose an Attention-based Spatial–Temporal Graph Neural Network (ASTGNN). Wang et al. [30] collected existing models to develop an open-source traffic benchmark models platform. Jiang et al. [31] designed a much more detailed model based on an improved attention mechanism PDFormer, which calculates attention scores with more information by using extra algorithms such as Dynamic Time Warping (DTW) [32] and k-shape clustering [33]. In summary, GAT-based models compute attention scores to dynamically update spatial–temporal information. Their incorporation of attention mechanisms has yielded promising results across diverse applications, showcasing their potential to enhance modeling capabilities in complex scenarios [34].

While CNN-RNN models excel in Euclidean feature extraction but struggle with graph-based spatial features. GCNs overcome this by updating spatial information dynamically, yet they rely on static representations and struggle with capturing temporal changes. GATs enhance modeling by using attention mechanisms to dynamically update spatial–temporal data, showing promise in complex scenarios. However, these approaches often neglect temporal nuances in traffic flow data, such as periodicity and chronological variations, which are critical for the comprehensive analysis of complex traffic features.

### 2.2. Multi-Granularity Temporal Embedding

Most of the related work on the traffic forecasting problem only takes the previous hour’s sample as the recent historical traffic input [7]. Existing studies have explored different granularity and chronological temporal embeddings. The GMAN [15] uses one-hot coding to encode the day-of-week and time-of-day of each time step into vectors. ASTGCN [28] intercepts three time series segments along the time axis as the input of the recent, daily period, and weekly period components, respectively. The three components share the same network structure and each of them consists of several spatial–temporal blocks and a fully connected layer. ASTGNN [29] embeds a global periodic sequence and a local periodic sequence to model traffic data’s periodicity. STEAFormer [35] focuses on input embedding and develops the periodicity embedding, including daily, weekly, and monthly. It denotes the learnable day-of-week embedding dict, and the timestamps-of-day embedding dict denotes the number of days in a week. In addition, STEAFormer designs a spatio-temporal adaptive embedding to capture intricate spatio-temporal relations uniformly.

Although these methods operate at different granularities [36,37], they have not fully utilized comprehensive temporal embedding information, leading to limitations in learning temporal features and hindering deeper extraction of latent characteristics. For instance, they have not effectively integrated information across various time scales, such as hourly, daily, and weekly periodic variations. The absence of integrated temporal embedding information may constrain the models’ capability to comprehend and forecast deeper features within traffic data. In contrast, our approach focuses on integrating different types of temporal embeddings that incorporate both periodicity and chronological aspects across varying granularities.

Moreover, these methods typically integrate only one type of temporal information at different granularities while ignoring positional temporal features, such as the specific day of the week to which each day belongs. Human activities often follow a weekly rhythm, which is reflected in traffic patterns that vary significantly between weekdays and weekends. Traffic flow data exhibit distinct characteristics depending on the day of the week, a critical feature overlooked by most existing methods even when incorporating multi-granularity temporal embeddings. For example, while daily or weekly data provide trends that can help the model learn comprehensive information, they fail to differentiate between weekdays and weekends or to distinguish the temporal position of a specific date within a week. This inability to account for such nuanced temporal characteristics limits the models’ capacity to extract additional data features that could further enhance their understanding of traffic patterns.

In contrast, MGTEFormer emphasizes the integration of different types of temporal embeddings that account for both periodicity and chronological aspects across varying granularities. Specifically, we design a multi-scale temporal embedding framework that captures the fine-grained temporal nuances of traffic patterns while simultaneously learning broader trends. By incorporating embeddings that encode distinct periodic behaviors, such as hourly, daily, and weekly cycles, alongside chronological sequences, our method enables the model to build a holistic understanding of temporal dependencies. Furthermore, our model explicitly considers the positional temporal features, such as the specific day of the week, to differentiate between weekdays and weekends and to recognize the temporal position of each date within the weekly cycle. This integrated approach ensures that both short-term irregularities and long-term patterns are effectively captured, enhancing the model’s ability to extract deeper latent features and deliver more accurate and reliable traffic flow predictions.

## 3. Methodology

### 3.1. Preliminary

The traffic flow forecasting task aims to predict the traffic flow in a certain period according to the historical traffic flow. Traffic flow prediction is a challenging problem in the field of intelligent traffic management [38]. The goal is to predict how the number of vehicles at observation points (sensors set up on highways) will change over time. The time interval is usually set to 5, 15, or 30 min.

In general, traffic flow is the average number of vehicles observed on that section of road over a given period. $X_{t}\in R^{N\times C}$ is used to represent the traffic flow of *N* sensors in the road network at time *t*, where *C* is the dimension of traffic flow, including traffic flow samples of different granularity, time and location information. Then, $X=\left(X_{1},\;X_{2},\;\ldots ,\;X_{T}\right)\in R^{T\times N\times C}$ represents the traffic flow information of all sensors in *T* time slices.

The purpose of traffic flow forecasting is to predict the traffic flow of a traffic system in the future time given historical observations. Formally, given the traffic flow tensor *X* observed on the traffic system, the traffic flow prediction problem learns the mapping function f from the observed flow value of the previous *T* time steps to predict the traffic flow of the future *T* time steps, which is shown in Equation (1).

(1) $$\left[X_{t-T+1},\;\ldots ,\;X_{t}\right]\overset{f}{\to }\left[X_{\left(t+1\right)},\;\ldots ,\;X_{\left(t+T^{\prime }\right)}\right].$$

### 3.2. Model Architecture

We design two Transformer encoders to capture the dynamic spatial–temporal dependencies. A temporal encoder module to capture the dynamic and inherent temporal patterns. A spatial encoder module to capture complex spatial information on different sensors. The two encoders comprise three similar primary components: a scaled dot-product multi-head attention network, a feed-forward network, and a residual network.

**Figure 1:**
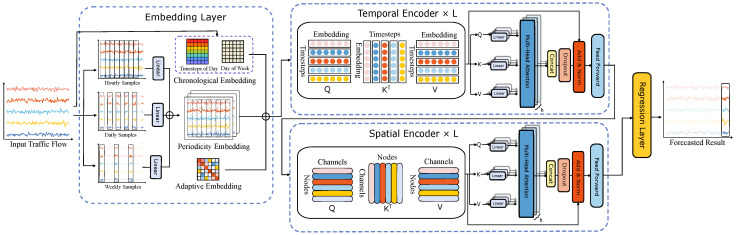
The framework of the proposed MGTEFormer. The model integrates a multi-granularity temporal embedding module to process time-series data, a Transformer-based spatio-temporal feature extraction module to capture complex patterns, and a prediction module with fully connected layers for output.

Our proposed model MGTEFormer builds on the general Transformer encoder framework, and Figure 1 shows its overall architecture. The encoder consists of a stack of identical layers. In order to support effective training as the model increases in depth, the residual connection and layer normalization are used inside the blocks, and all layers in the encoder maintain the same dimensionality.

Figure 1 shows the overall architecture of the multi-granularity temporal embedded Transformer model. The model consists of three parts, which are a successively multi-granularity temporal embedding module, a Transformer spatio-temporal feature extraction module, and a regression prediction output module. At the beginning, the multi-granularity time embedding module samples the original input data in the direction of the time axis to obtain different granularity data, and generates corresponding timing embedding to describe the relative position of the original input data in different time granularity periods. Secondly, complex spatio-temporal features are extracted through the self-attention mechanism in the temporal attention module and the spatial attention module. Finally, the extracted features are used to predict the traffic flow through two fully connected layers.

### 3.3. Multi-Granularity Temporal Embedding

To acquire the different granularity of traffic flow data, we took three different types of samples from the origin traffic flow data: previous hour samples, previous day samples, and previous week samples. These data samples are from historical data with predicted target $X_{t:T}$ as the reference object.

The previous hour sample is defined as $X_{hour}=FC\left(X_{t-T-hour+1:t-hour}\right)$, where FC represents a linear project layer and $X_{hour}\in R^{T\times N\times d_{h}}$. The previous day sample is defined as $X_{day}=FC\left(X_{t-T-day+1:t-day}\right)$, where FC represents a linear project layer and $X_{day}\in R^{T\times N\times d_{d}}$. The previous week sample is defined as $X_{week}=FC\left(X_{t-T-week+1:t-week}\right)$, where FC represents a linear project layer and $X_{week}\in R^{T\times N\times d_{w}}$. These three embeddings are concatenated, forming the output $X_{periodic}\in R^{T\times N\times d_{p}}$ the periodic embedding:

(2) $$X_{\mathrm{periodic}\;}=X_{\mathrm{hour}}\;\left|\right|X_{\mathrm{day}}\;∥X_{\mathrm{week}}.$$

where the dimension $d_{p}$ is equal to $d_{h}+d_{d}+d_{w}$. Additionally, to account for both weekly and daily periodicities and avoid periodic disturbances, the chronological embedding $X_{chrono}\in R^{T\times N\times d_{c}}$ is produced. This is achieved by referencing the day-of-week and timestep-of-day data to extract two distinct embeddings, which are subsequently concatenated and broadcasted to generate the chronological embedding with dimension $d_{c}$.

Day-of-week and timestep-of-day represent two temporal features. The day-of-week indices, ranging from 1 to 7, are derived by truncating from the date index. Similarly, timestep-of-day indices, ranging from 1 to 288, correspond to time intervals within a day, each representing a 5-min interval. Normalizing the time intervals of timestep-of-day enables the generation of standardized embeddings for both the day of week and timestep of day, yielding values within the range of 0 to 1. The day of week embedding is $X_{dow}\in R^{T\times N\times 1}$. The timestep of day embedding is $X_{tod}\in R^{T\times N\times 1}$. These embedding values are normalized based on the underlying data distribution.

(3) $$X_{\mathrm{chorno}}\;=X_{\mathrm{dow}}\;∥X_{\mathrm{tod}}.$$

Furthermore, to address diverse temporal patterns specific to each sensor, an adaptive embedding of dimension $d_{a}$ represented as $X_{adp}\in R^{T\times N\times d_{a}}$ is introduced to capture diverse temporal patterns for each sensor. Dynamic sensor time information $T_{dynamic(t)}$ is learned dynamically in the network through sensor embedding information.

These three embeddings are concatenated, forming the output $X_{embedding}\in R^{T\times N\times d_{embed}}$ the final embedding:

(4) $$X_{\mathrm{embedding}}\;=X_{\mathrm{periodic}}\;\left|\right|X_{\mathrm{chrono}}\;∥X_{\mathrm{adp}}.$$

where the dimension $d_{embed}$ is equal to $d_{p}+d_{c}+d_{a}$.

### 3.4. Transformer Encoder

We design two Transformer encoders to capture the dynamic spatial–temporal dependencies. The temporal encoder module captures the dynamic and inherent temporal patterns, while the spatial encoder module captures complex spatial information on different sensors. The two encoders comprise three similar primary components: the scaled dot-product multi-head attention network, feed-forward network, and residual network.

#### 3.4.1. Temporal Encoder Layer

The temporal encoder analyzes the input embedding from the embedding layer. The core of the encoder layer is the self-attention mechanism. Queries Q, keys K, and values V are three basic elements in the self-attention mechanism, which are obtained by nonshared linear transformations from the input embedding:

(5) $$Q_{t}=K_{t}=V_{t}=FC\left(X_{\mathrm{embedding}}\right).$$

The multi-head self-attention first linearly projects the queries, keys, and values into different representation subspaces and then performs the attention function

(6) $$Q_{t}=\;{\mathrm{head}}_{1}⊕\;{\mathrm{head}}_{2}⊕\;{\mathrm{head}}_{3}\ldots ⊕\;{\mathrm{head}}_{n},$$

where ⊕ represents concatenation operation.

Self-attention networks represent a highly effective approach for capturing long-range temporal relationships among different time steps, with the most prominent example being the Transformer model. The scaled dot-product network is the core part of Transformers, in which the attention calculation is formulated as:

(7) $$A_{t}=\left(\frac{Q_{t}K_{t}^{T}}{\sqrt{d_{k}}}\right),$$

where $d_{k}$ denotes the scaling factor, whose value is equal to the dimension of the model. In the temporal encoder module, we calculate the attention score between each timestep to capture temporal dependencies. Finally, we can obtain the output of the self-attention module by multiplying the attention scores with the value matrix as

(8) $$Attention\left(K_{t},Q_{t},V_{t}\right)=\mathrm{Softmax}\left(A_{t}\right)V_{t}.$$

#### 3.4.2. Spatial Encoder Layer

The spatial encoder layer is similar to the temporal encoder module. To model the dynamic spatial dependencies, we adjust the dimension of computational attention to be between different sensors based on the output of the temporal encoder module. Three primary components K, Q, and *V* are linearly projected on the temporal attention:

(9) $$Q_{s}=K_{s}=V_{s}=FC\left(Attention\left(K_{t},\;Q_{t},\;V_{t}\right)\right),$$

(10) $$Q_{s}=\;{\mathrm{head}}_{1}⊕\;{\mathrm{head}}_{2}⊕\;{\mathrm{head}}_{3}\ldots ⊕\;{\mathrm{head}}_{n},$$

where ⊕ represents concatenation operation. The detail spatial attention can be defined as:

(11) $$A_{s}=\left(\frac{Q_{s}K_{s}^{T}}{\sqrt{d_{k}}}\right),$$

(12) $$Attention\left(K_{s},\;Q_{s},\;V_{s}\right)=\mathrm{Softmax}\left(A_{s}\right)V_{s}.$$

In this way, the spatial encoder module incorporates temporal attention and spatial attention between various sensors.

### 3.5. Output Layer

We use the Feedforward network to generate the predicted flow sequence. It combines a fully connected neural network.

(13) $$Y=FC(Attention\left(K_{s},\;Q_{s},\;V_{s}\right))$$

We use the Huber loss [39] to calculate the model loss. The Huber loss is less sensitive to outliers than the squared error loss, and the operation process is shown in Equation (14).

(14) $$Loss\left(Y,\hat{Y}\right)=\left\{\begin{array}{cc} \frac{1}{2}{\left(Y-\hat{Y}\right)}^{2},\; & \;|Y-\hat{Y}|\leq \delta \\ \delta |Y-\hat{Y}|-\frac{1}{2}\delta^{2},\; & \;\mathrm{Otherwise} \end{array}\right.$$

where *Y* denotes the real traffic flow value, and $\hat{Y}$ is the predicted traffic flow value of the model. δ is used to control the range of squared error loss in Huber loss.

## 4. Experiments

### 4.1. Datasets

Our MGTEFormer is verified on two traffic forecasting benchmarks, PEMS04 and PEMS08, which are from the California Transportation Performance Measurement System (PEMS) [28]. The traffic data in the two datasets are aggregated at an interval of 5 min, which means there are 12 frames in each hour. The task is to use traffic flow from the past hour to predict the flow from the next hour, which represents how we use 12 historical frames to predict 12 frames. There are 12 frames of traffic data per hour and 288 frames of traffic data per day. The sliding window is set to 12 to generate training samples from the original data set. The ratio of training set, verification set, and test set is 6:2:2. The detailed data set information is shown in Table 1.

The spatial configurations in the dataset are derived from the PEMS dataset, where sensors are strategically distributed across eight highways in San Bernardino, Southern California, with intervals typically ranging from 0.5 to 2 miles. These configurations align with prior widely-cited works [35,40], allowing for direct comparability. While the primary focus of this study is on temporal feature learning, spatial correlations are modeled using spatial attention mechanisms that dynamically learn dependencies among sensors.

**Figure 2:**
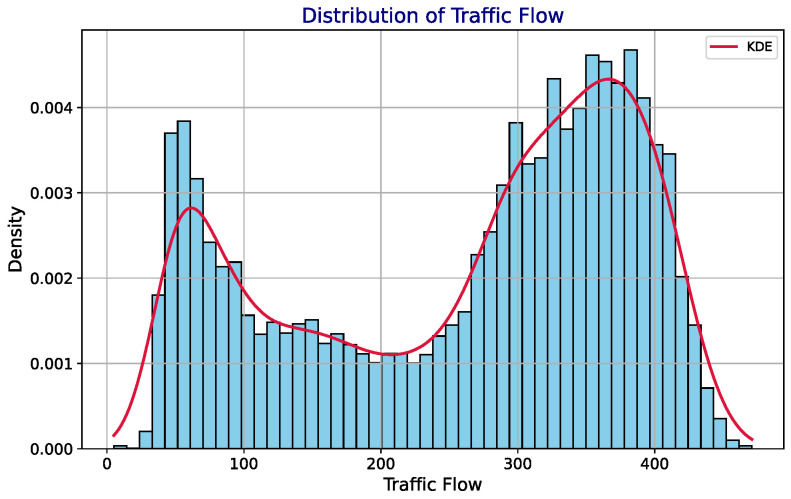
Traffic flow of PEMS08 at 23 different sensors. The histogram displays the distribution of traffic flow values, with the x-axis representing the traffic flow magnitude, and the y-axis depicting the density of these values. The Kernel Density Estimation (KDE) curve, illustrated in red, provides a smooth approximation of the underlying probability density function, highlighting two prominent peaks. The first peak around 100 units suggests a common traffic flow during off-peak hours, while the second peak near 350 units corresponds to peak traffic periods.

**Figure 3:**
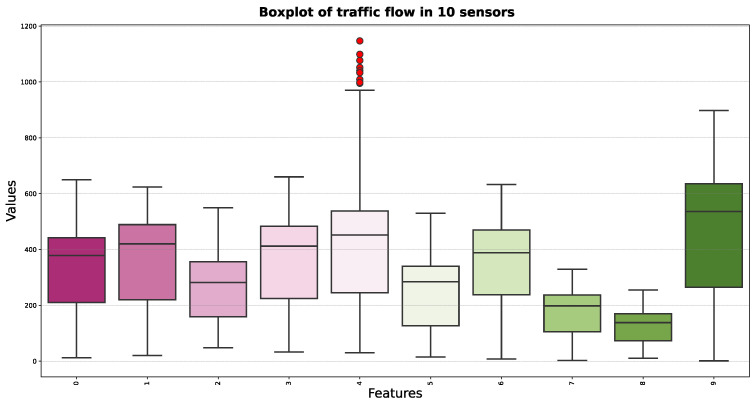
Traffic flow of PEMS08 at 10 different sensors. This boxplot visually represents the distribution of traffic flow values for 10 distinct sensors, with the x-axis denoting each sensor and the y-axis illustrating the range of traffic flow values. Each box illustrates the quartiles of the data, median, and spread, while whiskers extend to the most extreme data points within 1.5 times the interquartile range. Red dots represent outliers that fall beyond the whiskers, indicating unusual traffic flow values that deviate significantly from the typical distribution pattern.

Figure 2 presents a histogram of traffic flow data from 23 PEMS08 sensors, showing a bimodal distribution with peaks around 100 and 350 vehicles per minute. The red KDE curve smooths the distribution, confirming the two peaks, which likely correspond to different traffic intensity periods. This pattern is vital for traffic forecasting and management, indicating the need for models that can adapt to varying traffic volumes throughout the day. Figure 3 offers a boxplot of traffic flow variability across 10 PEMS08 sensors. The boxes and whiskers illustrate the spread and central tendency of the data, with outliers indicated by dots. The wide range and high median in the green box suggest greater traffic volume and variability, while the narrow pink box indicates less fluctuation and lower traffic.

To manage noise, we use robust scaling methods to normalize the data, reducing the influence of noise on the model’s learning process. For data points with zero values, we apply a masking technique to prevent these instances from skewing the training. As for outliers, such as those indicating traffic jams or accidents, we retain them, as they represent real-world events that our model must learn to handle.

**Figure 4:**
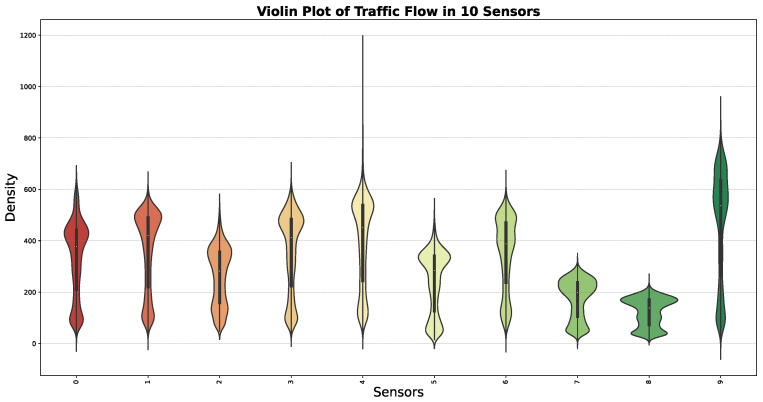
Traffic flow data violin plot of PEMS08 at 10 different sensors. The x-axis lists the sensors, and the y-axis shows traffic flow values. Each violin shape reveals the distribution and density of traffic flow, highlighting medians, quartiles, and data spread.

Figure 4 visualizes the distribution and density of traffic flow data across 10 PEMS08 sensors. The plot combines box plot elements with kernel density estimation to show the data’s distribution shape and concentration. Each sensor’s traffic flow data is represented by a violin shape, with wider sections indicating higher data density. The plot reveals that some sensors have a more concentrated traffic flow pattern, while others show a wider spread, indicating variability. The sensor with the green violin shape has a notably higher density at certain traffic flow values, suggesting a distinct traffic pattern compared to others.

**Figure 5:**
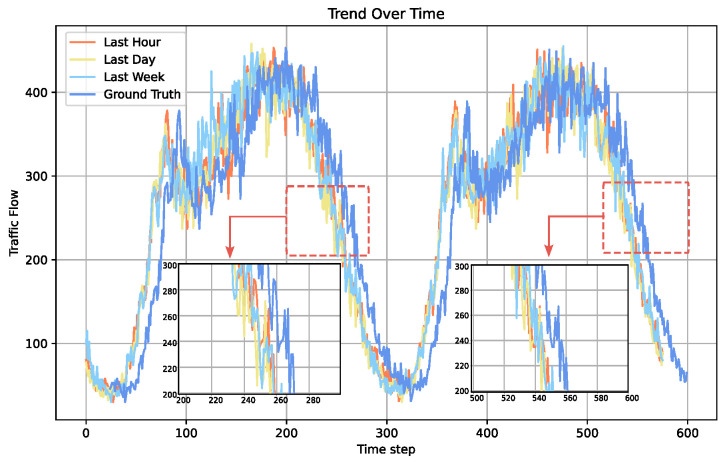
Traffic flow data distribution of PEMS08 sensor ID# 23 under different time granularity. The x-axis represents time steps, and the y-axis shows traffic flow. Orange tracks the last hour, yellow the previous day, light blue the last week, and dark blue represents the ground truth. The close alignment of these lines with the dark blue prediction line suggests a strong dependency on temporal granularity, indicating that the model’s predictions are closely aligned with historical data, capturing the periodicity and trend of traffic flow.

Spatio-temporal data have many types of temporal characteristics. Traffic flow is not only related to the most recent traffic flow but also has similarities to traffic flow at the same time on the day before or even the week before. As shown in Figure 5, multi-granularity traffic flow reflects the closeness, periodicity, and trend, respectively. Figure 5 illustrates the temporal dependencies captured in the traffic flow data across different time granularities: “Last Hour”, “Last Day”, “Last Week”, and “Ground Truth”. The “Ground Truth” represents the actual observed traffic flow data for the next hour relative to the historical data. The lines corresponding to “Last Hour”, “Last Day”, and “Last Week” depict the historical traffic patterns at different temporal scales, which serve as inputs for the model to generate predictions.

### 4.2. Experimental Setup

All experiments are conducted in PyTorch on an NVIDIA GeForce RTX 3090 GPU with 24 GB of memory. We configure the input and prediction lengths to 1 h, with $T=T^{\prime }=12$, and set the number of attention heads to 4. The Adam optimizer [41] is employed with an initial learning rate of 0.001, which is adjusted using a MultiStepLR scheduler with milestones set according to our configuration. The batch size is set to 16. To evaluate the model’s performance on traffic forecasting tasks, we use three standard metrics: Mean Absolute Error (MAE), Root Mean Squared Error (RMSE), and Mean Absolute Percentage Error (MAPE) [42]. These metrics are calculated based on the predictions and actual values to assess the accuracy of our model.

(15) $$MAE=\frac{1}{\epsilon }\sum_{i=1}^{\epsilon }\left|{\hat{Y}}^{i}-Y^{i}\right|$$

(16) $$RMSE=\sqrt{\frac{1}{\epsilon }\sum_{i=1}^{\epsilon }\left|{\hat{Y}}^{i}-Y^{i}\right|}$$

(17) $$MAPE=\frac{1}{\epsilon }\sum_{i}^{\epsilon }\frac{\left|{\hat{Y}}^{i}-Y^{i}\right|}{Y^{i}}\times 100\%$$

where $Y^{i}$ denotes the real traffic flow value and ${\hat{Y}}^{i}$ is the predicted traffic flow value of model. For each model, we regard the average performance of 12 horizons predicted as the final evaluated metrics. The data are standardized with Z-score normalization as $X^{\prime }=\frac{\left(X-X_{mean}\right)}{X_{std}}$, where $X_{mean}$ is the mean of the historical flow data, and $X_{std}$ is the standard deviation of the historical flow data.

### 4.3. Baselines

This study compares the MGTEFormer against baselines to validate its effectiveness. We consider STGNNs such as STGCN [14], DCRNN [43], ASTGCN [28], D2STGNN [44], and STWave [45]. Based on transformer models, we select GMAN [15], ASTGNN [4], PDFormer [31], and STAEformer [35], which are transformer models targeting the same task as ours.

### 4.4. Hyperparameter Tuning

In this section, we delve into a comprehensive analysis of critical hyperparameters: number of Transformer layers and attention heads. Our objective is to ascertain the optimal settings for these parameters through an empirical study, thereby enhancing the model’s performance. We will explore various configurations and evaluate their impact on the model’s predictive capabilities, aiming to identify the most effective parameter combinations automatically.

Table 2 provides a concise overview of the model’s performance across different configurations of Transformer layers and attention heads. The best results are observed with three Transformer layers and four attention heads, achieving a low MAE of 13.286, RMSE of 23.094, and a high R^2^ of 0.975. This configuration offers a balanced model complexity and generalization. The single-layer setup with four attention heads shows a higher MAPE, suggesting that additional layers may be necessary to effectively capture data complexity. Overall, the data support three layers and four heads as an optimal hyperparameter setting for the model.

All benchmark models were retrained using the dataset employed in this study to ensure consistency and fairness in comparison. For STGCN, the channels of the three layers in the ST-Conv block were set to 64, 16, and 64, respectively, with both the graph convolution kernel size *K* and temporal convolution kernel size $K_{t}$ set to 3. The initial learning rate was $10^{-3}$, with a decay rate of 0.7 after every 5 epochs. For STAEformer, the embedding dimensions were set to $d_{f}=24$ and $d_{a}=80$, with three layers for both spatial and temporal transformers and four attention heads. Models such as DCRNN, GMAN, PDFormer, and ASTGCN used the default settings specified in their original papers.

### 4.5. Results and Analysis

We present the comparison between different models on the PEMS08 and PEMS04 datasets. The overall performance comparison between different models is shown in Table 3 and Table 4. The compared results show that MGTEFormer outperforms all the baselines in MAE, RMSE, and MAPE metrics in PEMS08. MAE reflects the magnitude of the model’s average prediction error, RMSE considers the square of the error and gives higher weight to large errors, and MAPE reflects the model’s average percentage error. Our model demonstrates significantly improved performance in MAE, RMSE, and MAPE indicators compared to the baseline models. It achieves relatively low MAE, RMSE, and MAPE values, indicating its effectiveness in predicting traffic flow for both datasets. Additionally, it outperforms some other models in terms of certain metrics, such as ASTGCN and D2STGNN, especially on the PEMS04 dataset. For example, on the PEMS08 dataset, our model also showcases performance enhancements compared to the recent model STAEformer, and the three error indicators MAE, RMSE, and MAPE decreased by 0.23, 0.53, and 0.04, respectively. Overall, the experimental results indicate that our model exhibits the best performance.

**Figure 6:**
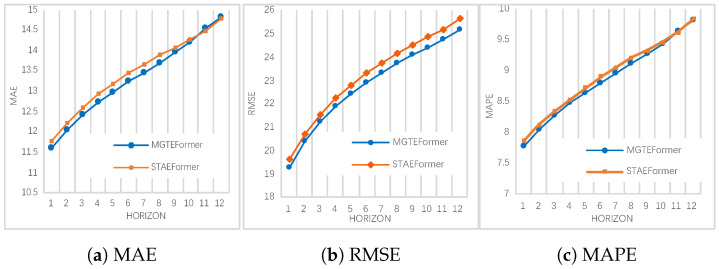
Forecasting performance comparison at each step on PEMS08. Subfigures (**a**) MAE, (**b**) RMSE, and (**c**) MAPE show the forecasting error metrics over a 12-step horizon. MGTEFormer (blue) consistently outperforms STAEformer (orange), indicating higher accuracy in predicting traffic flow.

Regarding the performance of single-step prediction, Figure 6 shows the evaluated metrics of each traffic flow in each horizon predicted. We plot visualization comparing the predicted traffic flow performance MAE, RMSE, and MAPE metrics in PEMS08 with the recent model STAEformer. We can see that the MGTEFormer model, which uses all five multi-granularity temporal embeddings, has the best MAE, RMSE, and MAPE metrics at almost every prediction step. As can be seen from the figure, the different error metrics all rise with increasing prediction step size, but the error for MATEFormer grows significantly less rapidly than the STAEformer.

**Figure 7:**
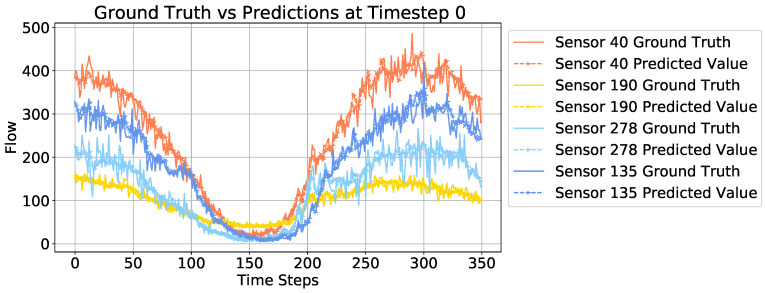
Forecasting performance comparison at timestep 0 on PEMS08. Solid lines depict actual traffic flow for four sensors, with dashed lines showing model predictions. Sensor 40 matches predictions closely, indicating high accuracy. Sensors 190 and 278 exhibit some prediction errors, suggesting refinement needs. Sensor 135 shows minor systematic deviations.

To illustrate the prediction results more vividly, we select some predicted traffic flow results from random sensors and compare them with the ground truth. The visualization is shown in Figure 7. The forecasting results on sensor 40, sensor 190, sensor 278, and sensor 135 are shown in Figure 7. It is obvious that the patterns of the four selected sensors are different. For example, there is often traffic congestion during the peak time at sensor 190, while sensor 40 often records traffic congestion during the start timesteps. This difficulty indicates that our model can capture these unique patterns for different sensors.

**Figure 8:**
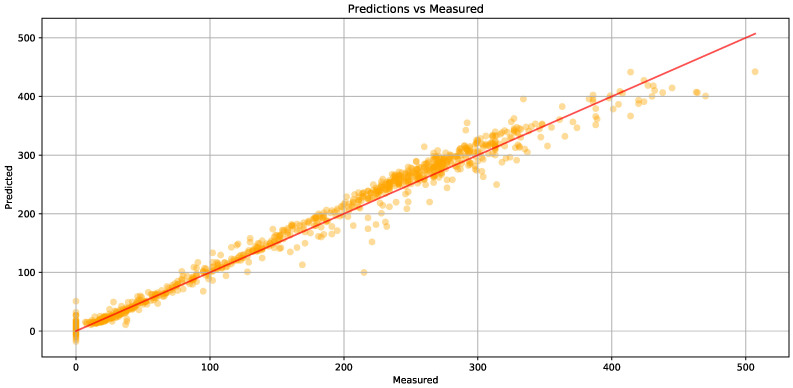
Plot of prediction vs. measurement of sensor 3 at step 0 in PEMS08 by MGTEFormer. Each dot represents a data sample, with the red line indicating perfect prediction alignment. The close clustering around the line shows a strong correlation, highlighting the model’s accuracy in traffic estimation.

**Figure 9:**
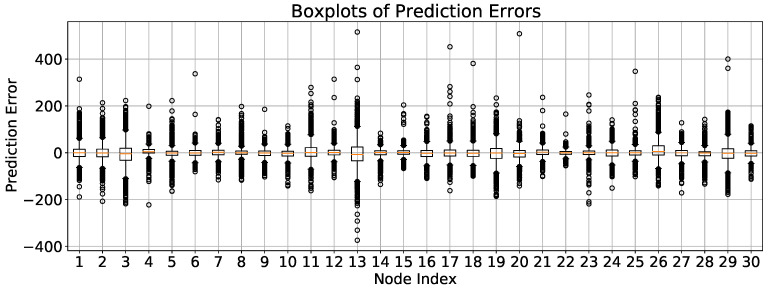
Boxplots of prediction errors of MGTEFormer in PEMS08 30 sensors. Boxplots illustrate the spread and median of prediction errors. The prediction errors are relatively small for most nodes, suggesting that the model generally performs well. However, a few nodes exhibit larger spread and more outliers, indicating less consistent performance. Notably, most nodes show relatively small prediction errors with medians near zero (indicated by the orange horizontal line in each box) which implies accurate predictions for those nodes.

Figure 8 illustrates the relationship between predicted and measured values in a research model. Each orange dot represents a data point, with the red line indicating the ideal prediction, where predicted values equal measured ones. The clustering of points around this line suggests a strong correlation, reflecting the model’s predictive accuracy. While some points deviate, indicating variance in prediction quality, the general pattern affirms the model’s reliability in forecasting. We randomly selected the data of 30 sensors for more detailed comparison, as shown in the Figure 9.

We evaluated the computational efficiency of the proposed MGTEFormer model by comparing it with several baseline models, focusing on two key metrics: average training time and inference time. The results, presented in Table 5, highlight that MGTEFormer achieves competitive training and inference times of 67.448 s and 9.320 s, respectively. When compared to other models, such as DCRNN and GMAN, MGTEFormer strikes an effective balance between computational efficiency and predictive accuracy. These baseline models, while delivering reasonable accuracy, often require significantly longer training and inference times, which may limit their practicality in real-time applications. Conversely, although models like PDFormer demonstrate faster training and inference times, their predictive performance does not match that of MGTEFormer. This trade-off underscores the strength of MGTEFormer in maintaining high accuracy while keeping computational costs manageable. The experimental results emphasize that MGTEFormer is well-suited for real-world scenarios, where both computational efficiency and predictive accuracy are critical. Its ability to provide robust predictions without compromising on speed makes it a versatile and practical choice for deployment in time-sensitive and resource-constrained environments.

**Figure 10:**
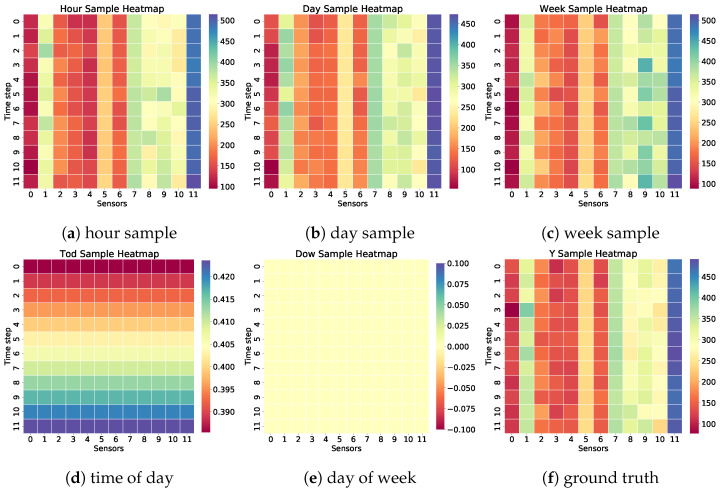
Visualization of the traffic flow of the multi-granularity sequence on PEMS08, including hour sample, day sample, week sample, time of day sample, day of week sample, and ground truth. Each heatmap uses color intensity to represent traffic flow magnitude, with darker shades indicating higher traffic volumes.

To underscore the significance of time-related data as an input parameter, we investigate the variations in different traffic granularities between the hour sample, day sample, week sample, time of day sample, and day of week sample. The traffic flow was gathered from 12 random sensors in the PEMS08 dataset over a one-hour duration. The first three samples represent three familiar sequences in different time granularities. Among these, the time of day sample and day of week sample represent the chronological position of each different specific time step. As illustrated in Figure 10, the horizontal axis delineates the 12 timesteps in an hour, the vertical axis represents 12 chosen sensors, and the color spectrum signifies varying traffic volumes. The multi-granularity sequence sample exhibited similar traffic flow patterns. Thus, incorporating different temporal information as model embedding proves advantageous for capturing the periodicity within the temporal sequence of traffic flow.

### 4.6. Ablation Studies

The ablation study was conducted on the PEMSD8 dataset to better understand the effect of different embeddings in MGTEFormer. The ablation study was divided into two parts: a comparison with the origin input [35] used in the previous work STAEformer, and a comparison with the single traffic flow concatenated with other embeddings. To better verify the effect of the multi-granularity temporal embedding we designed, we used each of the seven embeddings to input into the model: origin, which represents the traffic flow sequence concatenated with different temporal embeddings. The results of the ablation study on PEMS08 based on origin input are shown in Table 6. Conversely, a single traffic flow input was used as “x” as a model input. The ablation study on PEMS08 based on the single x input is shown in Table 7.

The numbers in parentheses indicate the total number of concatenated embeddings; origin(5) means the origin is concatenated from five multi-granularity temporal embeddings, the traffic flow sequence embedding, day sample embedding, week sample embedding, the day of week embedding, the timestep of day embedding; origin(3) means the origin is concatenated from three multi-granularity temporal embeddings, the traffic flow sequence embedding, and two other embeddings except the adaptive temporal–spatial embedding adp.

The different granularity temporal embedding: adp, which represents an adaptive temporal–spatial embedding [35]; day, which represents day sample embedding; week, which represents week sample embedding; dow, which represents the day of week embedding; tod, which represents the timestep of day embedding; ⊕, which represents the concatenate operation. The single x concatenated day-of-week embedding, timestep of day embedding, day sample embedding, week sample embedding, and adaptive temporal–spatial embedding outperform all embedding combinations.

## 5. Discussion

This section further examines the advantages and limitations of the MGTEFormer model in various traffic flow forecasting scenarios. The MGTEFormer model has been extensively evaluated for its efficacy and limitations in various traffic flow prediction contexts. Drawing from the empirical evidence discussed earlier, we arrive at the following conclusions: (1) Comparative analysis of diverse datasets reveals that the incorporation of multi-granularity embeddings has significantly enhanced predictive performance. Moreover, utilizing traffic data from the preceding hour rather than a composite origin input effectively minimizes noise and redundancy. (2) Incorporating inputs at various scales and applying chronological embeddings can significantly enhance the representation of trends across different time slices. The MGTEFormer is particularly effective at capturing complex, nonlinear fluctuation patterns, offering a more nuanced understanding of data dynamics. (3) Despite the impressive predictive capabilities of the MGTEFormer, its generalizability may be constrained due to its reliance primarily on two datasets. Future research should encompass datasets that reflect a broader range of geographical and traffic condition variations. (4) In this study, while spatial correlations are inherently captured within the model architecture, the primary focus is on the investigation of multi-granularity temporal embedding. This deliberate choice is due to the complexity of the interconnected highway network structure, which presents challenges in simultaneously exploring both spatial and temporal features. The spatial configuration follows well-established benchmarks, such as STAEformer and STGCN, ensuring consistency with baseline models and enabling fair comparisons. By maintaining this standardized spatial setup, we can isolate and evaluate the effectiveness of the proposed temporal embedding innovations without introducing additional spatial variables. The model demonstrates deficiencies in processing spatial features. Future iterations should focus on integrating more comprehensive spatial information, such as geographical data or traffic network topologies to improve spatial representation. These will include investigations of spatial correlations using specific location datasets, analysis of the impact of sensor placement on prediction accuracy, and the integration of road network topology information.

## 6. Conclusions

The proposed MGTEFormer model demonstrates promising capabilities in traffic flow prediction. Specifically, multi-granularity input embedding enables a more effective dynamic fusion of spatial-temporal features. The periodicity embedding and the chronological embedding describe the different characteristics of traffic flow data in different aspects. We then introduce a simple yet novel model, MGTEFormer, based on the attention mechanism. The multi-head attention mechanism extracts both localized and globalized features of the temporal dimension. We learned the complicated spatial and temporal dependencies by calculating the attention score between different sequences. We evaluated the performances of the MGTEFormer on traffic flow data from Caltrans PEMS and Washington. The decrease in different loss results proves the validity of our MGTEFormer. Moving forward, future efforts will be directed toward developing spatial features.

## Figures and Tables

**Table 1:** Dataset statistics.

Dataset	Samples	Sensors	Interval	Missing Ratio	Time Range
PEMS04	16,992	307	5 min	3.182%	1 January 2018–28 Fabruary 2018
PEMS08	17,856	170	5 min	0.696%	1 July 2016–31 August 2016

**Table 2:** Model performance of different hyperparameters.

Block Layers	Attention Heads	MAE	RMSE	MAPE	R2
3	1	13.205	22.912	8.842%	0.976
3	2	13.294	22.957	8.888%	0.975
3	4	13.286	23.094	8.816%	0.975
3	8	13.212	22.914	8.851%	0.976
1	4	13.625	22.808	9.492%	0.976
3	4	13.286	23.094	8.816%	0.975
5	4	13.234	22.997	8.789%	0.975
7	4	13.256	23.003	8.832%	0.975

**Table 3:** Model performance on PEMS08 and PEMS04 for traffic flow forecasting.

Dataset	Model	Metrics		
		MAE	RMSE	MAPE
PEMS08	STGCN	21.82	32.96	13.22%
	DCRNN	17.82	28.12	11.28%
	ASTGCN	18.18	28.05	11.00%
	GMAN	16.49	25.91	12.00%
	ASTGNN	16.02	25.51	10.09%
	D2STGNN	14.39	23.82	9.30%
	STWave	13.83	23.54	9.26%
	PDFormer	13.66	23.55	9.07%
	STAEformer	13.54	23.41	8.90%
	MGTEFormer	13.31	22.88	8.86%
PEMS04	STGCN	22.38	34.54	14.92%
	DCRNN	20.31	32.14	13.97%
	ASTGCN	21.89	34.53	15.00%
	GMAN	19.47	30.73	14.07%
	ASTGNN	18.77	31.04	12.44%
	D2STGNN	18.58	30.19	12.43%
	STWave	20.21	32.70	13.40%
	PDFormer	18.33	29.97	12.10%
	STAEformer	18.22	30.05	12.12%
	MGTEFormer	18.26	30.28	12.09%

**Table 4:** Horizon performance on PEMS08.

H	T	Metrics	STGCN	DCRNN	ASTGCN	GMAN	PDFormer	STAEformer	MGTEFormer
3	15 min	MAE	14.10	13.66	15.24	14.22	13.08	12.67	12.40
		RMSE	22.03	21.26	24.06	22.15	21.17	21.65	21.28
		MAPE	9.17%	8.85%	10.57%	10.54%	8.65%	8.24%	8.25%
		R2	0.977	0.979	0.973	0.977	0.979	0.978	0.979
6	30 min	MAE	15.37	14.45	15.84	14.63	13.75	13.54	13.21
		RMSE	23.97	22.71	25.16	23.05	22.50	23.48	23.01
		MAPE	9.85%	9.29%	10.63%	10.73%	9.11%	8.77%	8.78%
		R2	0.973	0.976	0.971	0.975	0.976	0.974	0.975
9	45 min	MAE	16.51	15.07	16.21	15.01	14.40	14.23	13.86
		RMSE	25.67	23.81	25.90	23.79	23.62	24.81	24.18
		MAPE	10.48%	9.94%	10.79%	10.98%	9.54%	9.21%	9.24%
		R2	0.969	0.973	0.969	0.973	0.973	0.971	0.973
12	60 min	MAE	17.56	15.60	16.63	15.39	15.15	14.94	14.54
		RMSE	27.25	24.71	26.59	24.45	24.76	25.99	25.21
		MAPE	11.06%	9.94%	10.99%	11.28%	10.05%	9.71%	9.73%
		R2	0.969	0.971	0.968	0.972	0.971	0.968	0.970

**Table 5:** Computational efficiency comparison.

Model	Average Training Time	Average Inference Time
DCRNN	282.233 s	38.653 s
GMAN	121.428 s	11.914 s
ASTGCN	96.266 s	20.485 s
D2STGNN	100.984 s	13.841 s
PDFormer	49.177 s	6.178 s
STAEformer	97.191 s	4.357 s
MGTEFormer	67.448 s	9.320 s

**Table 6:** Ablation study on PEMS08 with origin input.

Embedding	MAE	RMSE	MAPE (%)
origin(5)	15.96	25.97	10.52
origin(5)⊕ adp	14.65	23.89	9.53
origin(3)⊕day⊕week⊕adp	15.30	24.09	9.61
origin(3)⊕dow⊕tod⊕adp	13.44	23.27	8.92
origin(5)⊕dow⊕tod⊕day⊕week⊕adp	13.31	23.12	8.85

**Table 7:** Ablation study on PEMS08 with single x input.

Embedding	MAE	RMSE	MAPE (%)
x	17.11	27.43	10.92
x⊕adp	15.21	23.99	9.87
x⊕day⊕week	15.71	25.6	10.14
x⊕dow⊕tod	15.16	26.11	9.92
x⊕day⊕week⊕adp	15.36	24.14	9.68
x⊕dow⊕tod⊕adp	13.38	23.18	8.94
x⊕dow⊕tod⊕day⊕week⊕adp	13.31	22.88	8.86

## Data Availability

Data are contained within the article.

## References

[1] Sun L., Liu M., Liu G., Chen X., Yu X. (2024). FD-TGCN: Fast and Dynamic Temporal Graph Convolution Network for Traffic Flow Prediction. Inf. Fusion.

[2] Xu Z., Lv Z., Chu B., Li J. (2023). Fast Autoregressive Tensor Decomposition for Online Real-Time Traffic Flow Prediction. Knowl.-Based Syst..

[3] Pu B., Liu J., Kang Y., Chen J., Yu P.S. (2024). MVSTT: A Multiview Spatial-Temporal Transformer Network for Traffic-Flow Forecasting. IEEE Trans. Cybern..

[4] Huo G., Zhang Y., Wang B., Gao J., Hu Y., Yin B. (2023). Hierarchical Spatio–Temporal Graph Convolutional Networks and Transformer Network for Traffic Flow Forecasting. IEEE Trans. Intell. Transp. Syst..

[5] Chen X., Wu S., Shi C., Huang Y., Yang Y., Ke R., Zhao J. (2020). Sensing Data Supported Traffic Flow Prediction via Denoising Schemes and ANN: A Comparison. IEEE Sens. J..

[6] Zhang S., Yu W., Zhang W. (2024). Interactive dynamic diffusion graph convolutional network for traffic flow prediction. Inf. Sci..

[7] Zhang W., Yu Y., Qi Y., Shu F., Wang Y. (2019). Short-Term Traffic Flow Prediction Based on Spatio-Temporal Analysis and CNN Deep Learning. Transp. A Transp. Sci..

[8] Williams B.M., Hoel L.A. (2003). Modeling and Forecasting Vehicular Traffic Flow as a Seasonal ARIMA Process: Theoretical Basis and Empirical Results. J. Transp. Eng..

[9] Guo J., Huang W., Williams B.M. (2014). Adaptive Kalman Filter Approach for Stochastic Short-Term Traffic Flow Rate Prediction and Uncertainty Quantification. Transp. Res. Part C Emerg. Technol..

[10] Han Z., Zhao J., Leung H., Ma K.F., Wang W. (2021). A Review of Deep Learning Models for Time Series Prediction. IEEE Sens. J..

[11] Cai P., Wang Y., Lu G., Chen P., Ding C., Sun J. (2016). A Spatiotemporal Correlative K-Nearest Neighbor Model for Short-Term Traffic Multistep Forecasting. Transp. Res. Part C Emerg. Technol..

[12] Hong W.C., Dong Y., Zheng F., Wei S.Y. (2011). Hybrid Evolutionary Algorithms in a SVR Traffic Flow Forecasting Model. Appl. Math. Comput..

[13] Li J., Zhang Z., Meng F., Zhu W. (2022). Short-Term Traffic Flow Prediction via Improved Mode Decomposition and Self-Attention Mechanism Based Deep Learning Approach. IEEE Sens. J..

[14] Yu B., Yin H., Zhu Z. Spatio-Temporal Graph Convolutional Networks: A Deep Learning Framework for Traffic Forecasting. Proceedings of the Twenty-Seventh International Joint Conference on Artificial Intelligence.

[15] Zheng C., Fan X., Wang C., Qi J. (2020). GMAN: A Graph Multi-Attention Network for Traffic Prediction. Proc. AAAI Conf. Artif. Intell..

[16] Zhang S., Guo Y., Zhao P., Zheng C., Chen X. (2022). A Graph-Based Temporal Attention Framework for Multi-Sensor Traffic Flow Forecasting. IEEE Trans. Intell. Transp. Syst..

[17] Daraghmi Y.A., Yi C.-W., Chiang T.-C. Space-Time Multivariate Negative Binomial Regression for Urban Short-Term Traffic Volume Prediction. Proceedings of the 2012 12th International Conference on ITS Telecommunications.

[18] Daraghmi Y.A., Yi C.-W., Chiang T.-C. (2014). Negative Binomial Additive Models for Short-Term Traffic Flow Forecasting in Urban Areas. IEEE Trans. Intell. Transp. Syst..

[19] Al-Huthaifi R., Li T., Al-Huda Z., Li C. (2024). FedAGAT: Real-time Traffic Flow Prediction Based on Federated Community and Adaptive Graph Attention Network. Inf. Sci..

[20] Kong J., Fan X., Zuo M., Deveci M., Jin X., Zhong K. (2024). ADCT-Net: Adaptive Traffic Forecasting Neural Network via Dual-Graphic Cross-Fused Transformer. Inf. Fusion.

[21] Fu R., Zhang Z., Li L. Using LSTM and GRU Neural Network Methods for Traffic Flow Prediction. Proceedings of the 2016 31st Youth Academic Annual Conference of Chinese Association of Automation (YAC).

[22] Wu Z., Pan S., Long G., Jiang J., Zhang C. (2019). Graph WaveNet for Deep Spatial-Temporal Graph Modeling. arXiv.

[23] Zhao L., Song Y., Zhang C., Liu Y., Wang P., Lin T., Deng M., Li H. (2020). T-GCN: A Temporal Graph ConvolutionalNetwork for Traffic Prediction. IEEE Trans. Intell. Transp. Syst..

[24] Liu T., Jiang A., Miao X., Tang Y., Zhu Y., Kwan H.K. (2021). Graph-Based Dynamic Modeling and Traffic Prediction of Urban Road Network. IEEE Sens. J..

[25] Jin G., Liang Y., Fang Y., Shao Z., Huang J., Zhang J., Zheng Y. (2023). Spatio-Temporal Graph Neural Networks for Predictive Learning in Urban Computing: A Survey. arXiv.

[26] Jiang W., Luo J. (2022). Graph Neural Network for Traffic Forecasting: A Survey. Expert Syst. Appl..

[27] Vaswani A., Shazeer N., Parmar N., Uszkoreit J., Jones L., Gomez A.N., Kaiser Ł., Polosukhin I. Attention Is All You Need. Proceedings of the 31st International Conference on Neural Information Processing Systems.

[28] Guo S., Lin Y., Feng N., Song C., Wan H. (2019). Attention Based Spatial-Temporal Graph Convolutional Networks for Traffic Flow Forecasting. Proc. AAAI Conf. Artif. Intell..

[29] Guo S., Lin Y., Wan H., Li X., Cong G. (2022). Learning Dynamics and Heterogeneity of Spatial-Temporal Graph Data for Traffic Forecasting. IEEE Trans. Knowl. Data Eng..

[30] Jiang J., Han C., Jiang W., Zhao W.X., Wang J. (2024). LibCity: A Unified Library Towards Efficient and Comprehensive Urban Spatial-Temporal Prediction. arXiv.

[31] Jiang J., Han C., Zhao W.X., Wang J. (2023). PDFormer: Propagation Delay-Aware Dynamic Long-Range Transformer for Traffic Flow Prediction. Proc. AAAI Conf. Artif. Intell..

[32] Berndt D.J., Clifford J. Using Dynamic Time Warping to Find Patterns in Time Series. Proceedings of the 3rd International Conference on Knowledge Discovery and Data Mining.

[33] Paparrizos J., Gravano L. (2016). K-Shape: Efficient and Accurate Clustering of Time Series. ACM SIGMOD Rec..

[34] Geng Z., Xu J., Wu R., Zhao C., Wang J., Li Y., Zhang C. (2024). STGAFormer: Spatial–Temporal Gated Attention Transformer Based Graph Neural Network for Traffic Flow Forecasting. Inf. Fusion.

[35] Liu H., Dong Z., Jiang R., Deng J., Deng J., Chen Q., Song X. Spatio-Temporal Adaptive Embedding Makes Vanilla Transformer SOTA for Traffic Forecasting. Proceedings of the 32nd ACM International Conference on Information and Knowledge Management.

[36] Gao M., Du Z., Qin H., Wang W., Jin G., Xie G. (2024). Dynamic multi-scale spatial-temporal graph convolutional network for traffic flow prediction. Knowl.-Based Syst..

[37] Li Y., Xu H., Zhang T., Li X., Li G., Tian W. (2024). DDGformer: Direction- and distance-aware graph transformer for traffic flow prediction. Knowl.-Based Syst..

[38] Tian J., Han L., Chen M., Xu Y., Chen Z., Zhu T., Sun L., Lv W. (2024). MFGCN: Multi-faceted spatial and temporal specific graph convolutional network for traffic-flow forecasting. Knowl.-Based Syst..

[39] Huber P.J., Kotz S., Johnson N.L. (1992). Robust Estimation of a Location Parameter. Breakthroughs in Statistics.

[40] Song C., Lin Y., Guo S., Wan H. (2020). Spatial-Temporal Synchronous Graph Convolutional Networks: A New Framework for Spatial-Temporal Network Data Forecasting. Proc. AAAI Conf. Artif. Intell..

[41] Kingma D.P., Ba J. (2017). Adam: A Method for Stochastic Optimization. arXiv.

[42] Chen J., Zheng L., Hu Y., Wang W., Zhang H., Hu X. (2024). Traffic Flow Matrix-Based Graph Neural Network with Attention Mechanism for Traffic Flow Prediction. Inf. Fusion.

[43] Li Y., Yu R., Shahabi C., Liu Y. (2018). Diffusion Convolutional Recurrent Neural Network: Data-Driven Traffic Forecasting. arXiv.

[44] Shao Z., Zhang Z., Wei W., Wang F., Xu Y., Cao X., Jensen C.S. (2022). Decoupled Dynamic Spatial-Temporal Graph Neural Network for Traffic Forecasting. Proc. VLDB Endow..

[45] Fang Y., Qin Y., Luo H., Zhao F., Xu B., Zeng L., Wang C. When Spatio-Temporal Meet Wavelets: Disentangled Traffic Forecasting via Efficient Spectral Graph Attention Networks. Proceedings of the 2023 IEEE 39th International Conference on Data Engineering (ICDE).

